# Renal injury in cardiorenal syndrome type 1 is mediated by albumin

**DOI:** 10.14814/phy2.15173

**Published:** 2022-02-12

**Authors:** Yoshio Funahashi, Mizuko Ikeda, Rumie Wakasaki, Sheuli Chowdhury, Tahnee Groat, Douglas Zeppenfeld, Michael P. Hutchens

**Affiliations:** ^1^ Anesthesiology & Perioperative Medicine Oregon Health & Science University Portland Oregon USA; ^2^ Operative Care Division Portland Veterans Affairs Medical Center Portland Oregon USA

**Keywords:** acute kidney injury, albumin, cardiac arrest and cardiopulmonary resuscitation, cardiorenal syndrome

## Abstract

Cardiorenal syndrome type 1 (CRS‐1) acute kidney injury (AKI) is a critical complication of acute cardiovascular disease but is poorly understood. AKI induces acute albuminuria. As *chronic* albuminuria is associated with worsening kidney disease and albumin has been implicated in tubular epithelial injury, we investigated whether albumin participates in CRS‐1, and whether CRS‐1 alters renal albumin handling. We report the role of albumin in *in vivo* and *in vitro* CRS‐1 models. An established translational model, cardiac arrest and cardiopulmonary resuscitation (CA/CPR) induced severe acute albuminuria which correlated with tubular epithelial cell death. *In vivo* microscopy demonstrated CA/CPR‐induced glomerular filtration of exogenous albumin, while administration of exogenous albumin after CA/CPR worsened AKI compared to iso‐oncotic control. Increased albumin signal was observed in the proximal tubules of CA/CPR mice compared to sham. Comparison of albumin flux from tubular lumen to epithelial cells revealed saturated albumin transport within minutes of albumin injection after CA/CPR. *In vitro*, HK2 cells (human kidney tubular epithelial cells), exposed to oxygen‐glucose deprivation were injured by albumin in a dose dependent fashion. This interference was unchanged by the tubular endocytic receptor megalin. In conclusion, CRS‐1 alters albumin filtration and tubular uptake, leading to increased tubular exposure to albumin, which is injurious to tubular epithelial cells, worsening AKI. Our findings shed light on the pathophysiology of renal albumin and may guide interventions such as albumin resuscitation to improve CRS‐1 outcomes. This investigation may have important translational relevance for patients that receive exogenous albumin as part of their CRS‐1 treatment regimen.

## Graphical abstract

Administered albumin worsens AKI in cardiorenal syndrome type 1; this is associated with increased albumin filtration and altered tubular albumin uptake.

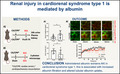

## 1 INTRODUCTION

Cardiorenal syndrome type 1 (CRS‐1) is characterized as acute kidney injury (AKI) due to rapid worsening of cardiac function (Ronco et al., 2008). It is common in patients following acute myocardial infarction (AMI) (29.9%), cardiac arrest (43%), or cardiothoracic surgery (43%) (Hobson et al., 2009; Sun et al., 2018; Tujjar et al., 2015). Patients with CRS‐1 suffer higher mortality than heart failure patients without AKI. In addition, CRS‐1 is associated with longer hospital stays and a higher risk of re‐admission (Damman et al., 2007). Unfortunately, the pathophysiology of CRS‐1 remains unclear, and there is no specific therapy. The translational mouse model of cardiac arrest and cardiopulmonary resuscitation (CA/CPR) induces profound CRS‐1 (Matsushita et al., 2020). Using this model, we previously found that the glomerular filtration barrier is altered by CA/CPR, which results in transient glomerular hyperpermeability to macromolecules (Hutchens et al., 2012). It is widely recognized that proteinuria and albuminuria in chronic kidney disease (CKD) are associated with the progression to end‐stage renal disease (Lambers Heerspink & Gansevoort, 2015). Therefore, we hypothesized that CRS‐1 related glomerular hyperpermeability induces increased albumin filtration, and this increased tubular albumin worsens AKI in CRS‐1. We investigated CA/CPR‐induced proteinuria and AKI as well as the dynamics of exogenous albumin filtration and tubular uptake after CA/CPR by 2‐photon microscopy. Finally, we performed *in vitro* testing of the mechanism in human kidney epithelial cells under oxygen‐glucose deprivation (OGD) with albumin‐containing media.

## 2 MATERIALS AND METHODS

### 2.1 Cardiac arrest and cardiopulmonary resuscitation in mice (CA/CPR)

All animal procedures were approved by the Oregon Health & Science University Institutional Animal Care and Use Committee and the VA Portland Health Care System Institutional Animal Care and Use Committee. Eight to twelve week‐old male C57BL/6 mice (obtained from Jackson Laboratories) underwent normothermic CA/CPR as previously described (Hutchens et al., 2012). Briefly, after weighing, anesthesia was induced with 4% isoflurane in a 2:1 air:oxygen mixture, followed by 1–2% isoflurane for maintenance. Mice were placed in a supine position on a heated pad, and using a rectal temperature probe, and lamp, temperatures were maintained between 36.5 and 37.5°C. After tracheal intubation, performed with a 22‐ga Teflon catheter (InSyte‐W, BD, Franklin, NJ), mice were mechanically ventilated with 150 uL tidal volume, 150 breaths per minute. Subcutaneous electrocardiography (EKG) leads were placed. The right jugular vein was catheterized with a PE‐10 tube and cardiac arrest (CA) was induced with the intravenous infusion of 20 μEq (40 μl) of 0.5 M potassium chloride. CA was confirmed by isoelectric EKG and absence of visible chest wall movement due to cardiac contraction. Upon confirmed CA, air, oxygen, and isoflurane were turned off. At the same time, mechanical ventilation was discontinued and disconnected from the endotracheal tube. Seven minutes and 30 s after CA, the endotracheal tube was reconnected with mechanical ventilation using 100% oxygen. Eight minutes after CA, chest compressions were initiated at a rate of 300 beats per minute. Up to 1.0 ml (16 μg) of epinephrine in 0.9% sodium chloride solution, was administrated via the jugular vein catheter. Return of spontaneous circulation (ROSC) was confirmed by EKG and visible cardiac contractions on the chest wall. Following ROSC confirmation, 5% albumin or the iso‐oncotic control, Ficoll‐70, was administrated via the jugular vein catheter. The dose of albumin was 0.3g/kg (weight‐equivalent to 500 ml of 5% albumin in a 70 kg human). The jugular catheter was removed. After the achievement of >60 breaths/min spontaneous respiration, the trachea was extubated. Animals were placed in a recovery cage, which was placed on a warming pad controlled at 37°C. For sham‐treated mice, general anesthesia was performed as described for CA/CPR, with endotracheal intubation, mechanical ventilation, and catheter placement.

### 2.2 Measurement of urine albumin

In a separate cohort of mice, 24‐h collection of urine was performed before and after CA/CPR. Urine albumin concentration was quantified by ELISA according to manufacturer instructions (Albuwell, Newtown Sq, PA).

### 2.3 Unbiased stereology measurement of tubular necrosis

Paraffin‐embedded kidney sections were stained with Fluoro‐Jade B as previously described to identify necrotic cells. Unbiased stereology was then used to determine the volume fraction of necrotic tubules (VNT) (Wakasaki et al., 2018, 2019). Briefly, a microcontrolled movable stage was used to assess systematic random high‐power sections for necrotic cells adjacent to systematically imposed grid points. The volume of necrotic tubules is calculated in reference to the total kidney volume determined using a separate, systematic random grid imposed on the whole kidney, as previously described (Gundersen et al., 1999).

### 2.4 Measurement of serum creatinine

Creatinine was quantified in serum samples using a creatinine aminohydrolase‐based autoanalyzer assay (Abaxis, Union City, CA).

### 2.5 Multiphoton renal imaging

Following CA/CPR or sham procedure, general anesthesia and the jugular catheter were maintained, and mice were subjected to *in vivo* microscopic imaging as described previously (Matsushita et al., 2018). A small skin incision was made on the left flank, and the kidney was partly extruded through the muscle and subcutaneous tissue. The exposed kidney was immobilized in 10% agar. An aluminum frame within a custom polysiloxane (Sugru, London, England) support was applied to support a cover glass, and any gaps were filled with 1% agar. The mouse was then immobilized on the imaging platform, and a heating pad was used to maintain normothermia. One hundred microliters (100 μl) FITC‐dextran (2000‐kDa molecular weight) and Alexa‐594‐(A594)‐conjugated BSA was injected via the jugular catheter immediately before imaging. Imaging was carried out using a Zeiss LSM 7MP microscope with 525/50 and 593/35 band‐pass filters and excitation set at 780 nm, with power between 20 and 40 μA. A single glomerulus was centered for optimal imaging within a 500‐micron window with surrounding tubules and a z‐stack 75 microns in depth was captured every 2 min for 60 min after FITC‐dextran injection. To quantify relative glomerular sieving of fluorescent albumin, fluorescence of albumin and dextran were quantified within the glomerular capillary tuft and within Bowman's urinary space. The ratio of capillary tuft:urinary space fluorescence (θ) was determined for albumin and dextran, and fluorescence of dextran (a non‐filtering control for off‐target fluorescence) was subtracted from that of albumin to yield the Δθ (albumin‐dextran).

### 2.6 Multiphoton‐derived assessment of albumin tubular transport

Dynamics of albumin transport at proximal tubules was quantified as albumin flux from tubular lumen to brush borders. The relative intensity of albumin was determined by the ratio of the fluorescence of albumin to dextran in the proximal tubular lumen (L) or brush borders and tubular cells (BB/C). Next, the ratio of albumin intensity of BB/C to total albumin intensity of the lumen and the tubular cell (L + BB/C) was determined every 2 min after the injection of BSA. They were then normalized by the value at the time of 5‐min. Three tubules with optimal imaging within the periglomerular z‐stack were evaluated in each mouse. Albumin flux was evaluated by curve fitting with non‐linear regression.

### 2.7 Human tubular epithelial cell culture

HK2 cells were purchased from American Type Culture Collection and cultured using standard HK2 media (10% fetal bovine serum in Dulbecco's Modified Eagle Medium and Hamm's F12, supplemented with D‐glucose [11 mM], gentamicin [106 μM], and recombinant human epidermal growth factor [807 pM]) in 6‐well plates. Cells were used for experiments when they were 80% confluent. To interfere with megalin expression, HK2 cells were transfected with *LRP2* siRNA (Thermo Fisher Scientific, Waltham, Massachusetts), *CUBN* siRNA (Thermo Fisher Scientific) or a scrambled negative control (Thermo Fisher Scientific) using Lipofectamine 3000 (Invitrogen, Carlsbad, CA). Briefly, culture medium was replaced to Opti‐MEM (Thermo Fisher Scientific) after rinsing with PBS, then mixture of Lipofectamine 3000 and 60 pg of siRNA was added to each well. After 24 h incubation, Opti‐MEM was changed to standard HK2 media. The sequences of siRNA are provided in Table S1. After siRNA interference, cells are incubated with 25 mg/ml albumin contained DMEM under oxygen and glucose deprivation or normo‐oxygen control condition for 16 h. Then cells were subjected to cell survival and death assays.

### 2.8 Oxygen‐glucose deprivation (OGD)

To conduct the OGD experiments, 12‐well plates containing cells were placed into an oxygen‐free chamber (Coy Lab Products, Grass Lake MI) with an oxygen monitor and palladium catalytic oxygen depletion system. Culture media was exchanged with pre‐equilibrated glucose‐free media containing bovine serum albumin or control. Oxygen concentration was maintained at 0 parts per million (monitored continuously with an oxygen monitor, Coy Lab Products, Grass Lake MI) for 16 h, after which cells were removed from the chamber and oxygen‐glucose repletion (with complete cell culture medium) was performed for 6 h prior to determining outcomes.

### 2.9 Cell survival and death assays

To determine cell survival the 3‐(4,5‐dimethylthiazol‐2‐yl)‐2,5‐diphenyltetrazolium bromide (MTT) assay was used. After OGD and oxygen‐glucose repletion, MTT reagent was added to wells. After 1 h incubation, the MTT reagent was aspirated, and then the cells were lysed by adding dimethyl sulfoxide (DMSO) to each well. Cell lysates were moved to 96 well plates to prepare for measuring absorbance at 540 nm. Cell survival was quantified as percent of normoxic/glucose replete control. To assess apoptosis, cells were stained with Hoechst 33342 (H33342, 1 μl per well) using a commercial kit (Invitrogen, Carlsbad, CA). Wells were imaged at high power (3 fields per well) for each stain on an inverted fluorescence microscope (ECLIPSE TE200, Nikon, Tokyo, Japan). Using ImageJ, images were set for identical thresholds and the number of cells positive for each stain were counted. Apoptotic cells were quantified as the number of H33342 stained cells with annexin V, and expressed as a proportion of the total number of H33342 stained cells. To quantify apoptosis, h33342‐stained images were examined, and apoptotic bodies (fragmented nuclei) were counted and expressed as a percent of total nuclei.

### 2.10 Immunoblotting

Kidney samples were homogenized in iced radioimmunoassay precipitation buffer with protease inhibitor (Complete, Roche Applied Science, Indianapolis, IN). The concentration of lysate protein was measured using the bicinchoninic acid assay. Twenty micrograms of protein was loaded into 4–12% gradient precast gels (NuPAGE Tris‐Acetate Gels, Invitrogen, Carlsbad, CA), electrophoresed, and transferred to polyvinyl difluoride membranes. After the Ponceau staining, blocking was performed using 5% skim milk. Membranes were then incubated in primary anti‐KIM‐1 antibody (R&D Systems, Minneapolis, MN) and HRP‐conjugated secondary antibody (R&D Systems). Blots were imaged using enhanced chemiluminescence (Thermo Fisher Scientific). The result was expressed as the ratio of the optical density of the Ponceau staining.

### 2.11 Statistical analysis

Statistical analysis was performed using GraphPad Prism 9.2.0 (GraphPad, LaJolla, CA). Student's *t*‐test was performed for the comparison of two‐group comparisons. ANOVA was performed for multiple‐group comparisons. Nonlinear regression was performed for the curve comparison. Statistical significance was inferred from *p* < 0.05. Mean and standard deviation are shown in the figures and text.

## 3 RESULTS

### 3.1 CRS‐1 induces albuminuria, and exogenous albumin worsens CRS‐1

**FIGURE 1:**
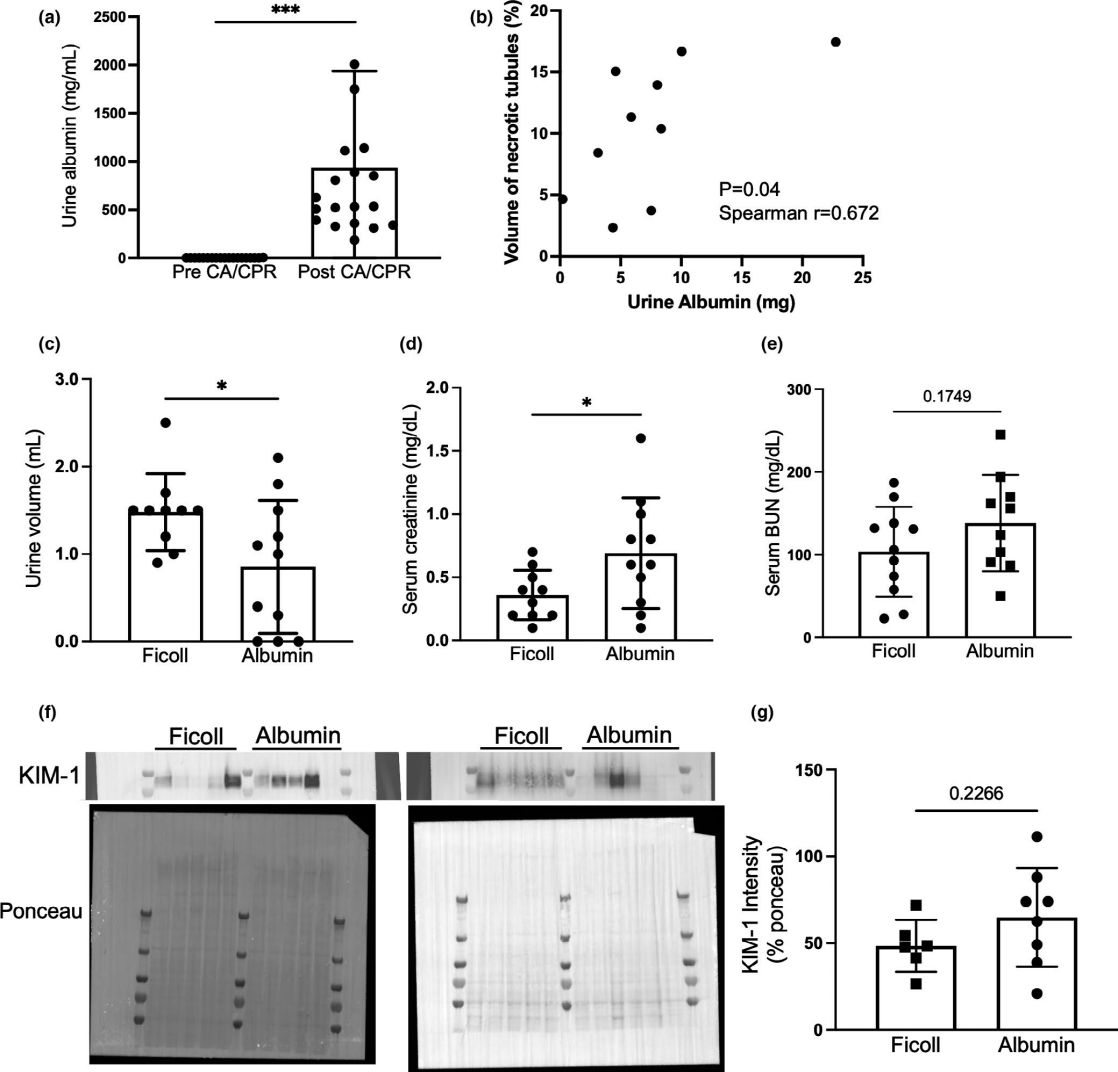
Albuminuria associates with worsened CRS‐1, and administered albumin worsens CRS‐1. (a) After CA/CPR, severe albuminuria was observed (*n* = 19/group). (b) Severity of albuminuria correlated with tubular epithelial cell death (VNT: volume of necrotic tubules, as measured by unbiased stereology) (*n* = 10/group). (c–e) Compared with Ficoll‐70 administration (iso‐oncotic control), albumin administration resulted in reduced urine output and increased serum creatinine 24 h after CA/CPR. BUN was not significantlydifferent (*n* = 10 in Ficoll control group, *n* = 11 in albumin group). (f and g) KIM‐1 expression in the kidney was not different between Ficoll and albumin group (*n* = 6–8/group). Statistical analysis is derived from Student's *t*‐test (a, c, d, e and g), or Pearson correlation analysis (b)

We observed severe albuminuria 24 h after CA/CPR (0.58 ± 0.18 mg/ml in pre‐CA/CPR vs. 934.1 ± 230.3 mg/ml in post‐CA/CPR, *p* < 0.0005, *n* = 19, Figure 1a). The severity of albuminuria was associated with tubular epithelial cell death (Pearson correlation coefficient *r* = 0.672, *p* = 0.039, *n* = 10, Figure 1b). Because of this association, we investigated whether albumin itself was injurious to the kidney in acute cardiorenal syndrome, compared with the iso‐oncotic control, Ficoll‐70. Compared with Ficoll, albumin resulted in reduced urine output (0.85 ± 0.23 ml vs. 1.48 ± 0.14 ml, *p* = 0.035, *n* = 10–11/group, Figure 1c), and doubled serum creatinine 24 h after CA/CPR (0.36 ± 0.06 mg/dl vs. 0.69 ± 0.13 mg/dl, *p* = 0.049, *n* = 10–11/group, Figure 1d). BUN was not significantly different between the groups, however (103.6 ± 54.2 mg/dl vs. 138.2 ± 58.2 mg/dl, *p* = 0.175, *n* = 10–11/group, Figure 1e). Twenty‐four hours after CA/CPR semi‐quantitation of renal kidney injury molecule‐1 (KIM‐1) by immunoblotting was not significantly different between kidney lysates from Ficoll‐70 and albumin‐treated mice (64.8 ± 28.5% vs. 48.4 ± 15.0%, *p* = 0.226, *n* = 10–11/group, Figure 1f and g) Taken together, these data indicate that albumnuria associates with worsened CRS‐1, and exogenous albumin administration worsens kidney injury due to acute cardiorenal syndrome.

### 3.2 CRS‐1 alters the kinetics of albumin filtration and reabsorption in the kidney

**FIGURE 2:**
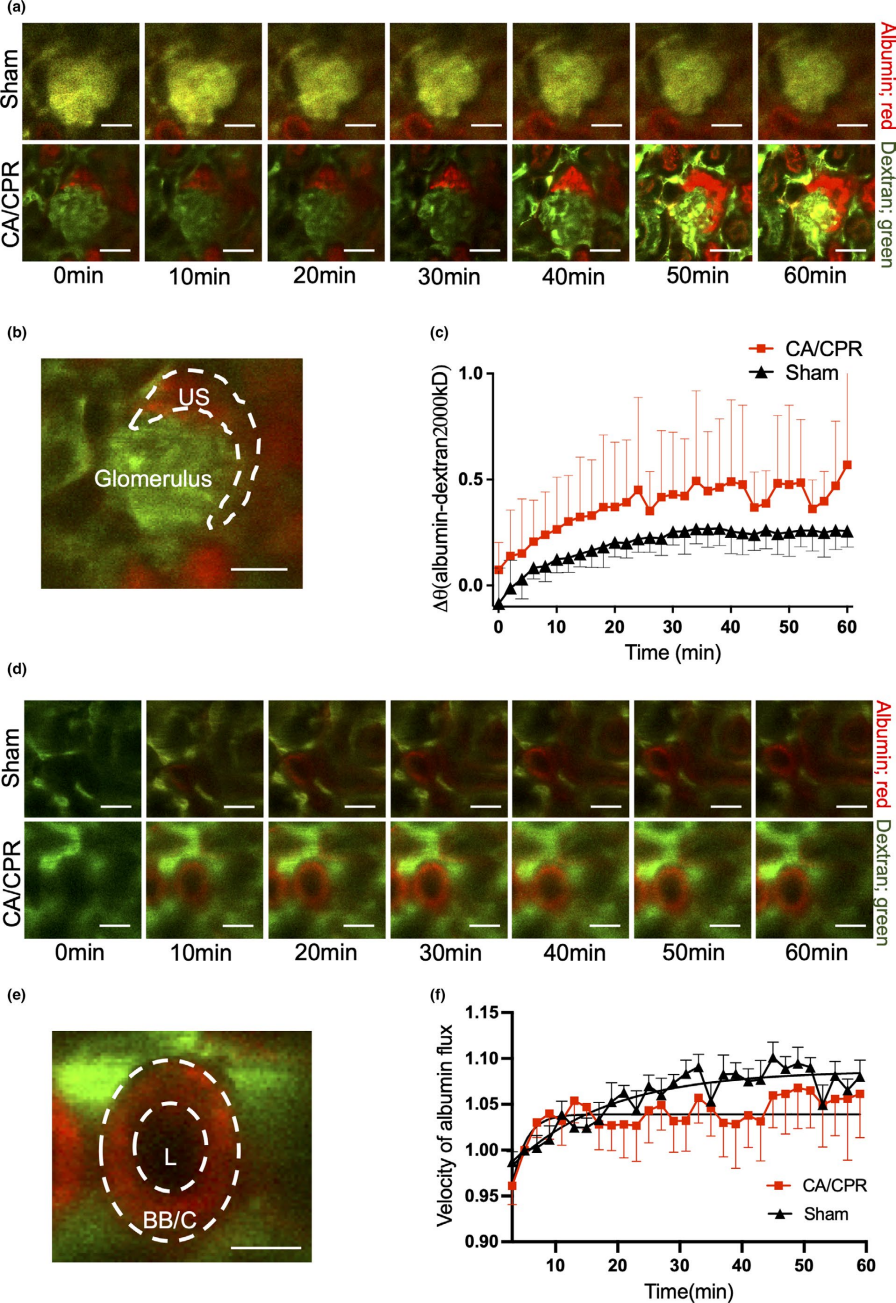
CA/CPR alters glomerular filtration and tubular flux of fluorescent albumin. (a) Higher intensity of albumin (red) was observed in the urinary space (US) of CA/CPR mice than sham. (b) Identification of the urinary space in the image of glomeruli. (c) CA/CPR caused increased relative urinary albumin over that of sham, implicating increased filtration of albumin due to CA/CPR within hours of the procedure, *p* < 0.05 by nonlinear regression. The rate of association and plateau of the curves were compared using the extra sum‐of‐squares F test. (d) 2‐photon images of proximal tubules at 0 h and 1 h of albumin administration. The white toroid is the region of interest used to quantify brush border and tubular cell fluorescence (BB/C), and the inner area of the small circle is the region of interest used to quantify fluorescence form the central lumen of the proximal tubules (L). Higher intensity of up‐taken albumin was observed in BB/C of CA/CPR mice than sham. (e) Identification of the lumen of the proximal tubules, and the brush border and the tubular cell. (f) Albumin flux from tubular lumen to tubular cell/brush borders is altered after CA/CPR. In sham, one‐phase association adequately modeled movement of luminal albumin to the brush border and cells with *R* ^2^ = 0.35. In CA/CPR, one‐phase association poorly modeled this albumin flux, and the fitted curve reached plateau much earlier, suggesting saturated albumin transport within minutes of albumin injection. Scale bar represents 50 µm. *N* = 5–6/group. Each data point is shown as mean ± SD

Next, we evaluated dynamic filtration of administered albumin in the kidney of mice with CRS‐1 by injecting Alexa 594 (A594)‐conjugated bovine serum albumin (BSA). Two‐photon microscopic images of glomeruli after A594‐BSA administration showed a higher intensity of albumin in the urinary space of CA/CPR mice compared with sham (Figure 2a and b). In images taken every 2 min, the quantified ratio of urinary space to capillary albumin fluorescence relative to that of non‐filtered, high molecular weight (2000 kD) dextran (Δθ) demonstrated increased albumin filtration in CA/CPR mice compared with sham (*p* = 0.001, *n* = 6/group, Figure 2c). In addition, we observed that albumin intensity at the brush border and within tubular cells in CA/CPR mice increased more quickly than in sham, followed by stabilized intensity in both group (Figure 2d and e). Non‐linear regression of albumin flux, the rate of change of the ratio between lumen albumin signal and that associated with the brush border and cells demonstrated different kinetics. One‐phase association adequately modeled the movement of luminal albumin to the brush border and cells with *R* ^2^ = 0.35 in sham, while in CA/CPR, one phase association poorly modeled this albumin flux, and the fitted curve reached plateau much earlier (*p* = 0.0002, *n* = 5–6/group, Figure 2f). These results demonstrate that glomeruli and tubular epithelial cells experience an altered transport kinetics of albumin in CRS‐1, and suggest that tubular albumin transport may rapidly reach saturation in acute cardiorenal syndrome.

### 3.3 Albumin causes dose‐dependent tubular cell death during modeled ischemia *in vitro*

**FIGURE 3:**
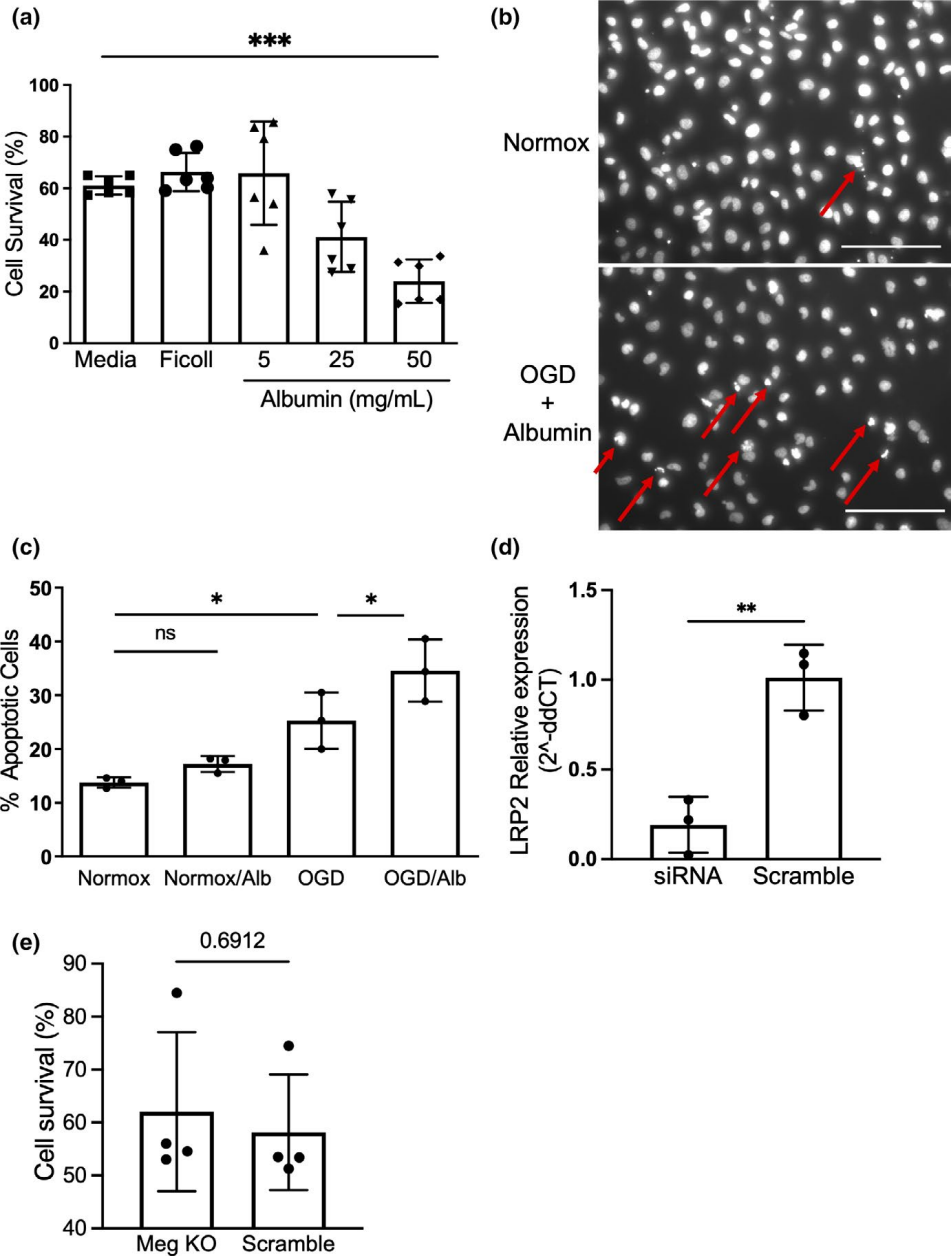
Albumin causes dose‐dependent HK2 cell death under OGD condition. (a) Albumin causes dose‐dependent reduction in cell survival in human tubular epithelial cells (HK2 cell line). HK2 cells received 16 h oxygen‐glucose deprivation (OGD) in control conditions or with increasing albumin concentrations. There was a dose dependent reduction in survival with albumin administration (*n* = 6/group, *p* < 0.0001). (b) An image of h33342‐stained HK2 cells. Red arrows indicate representative apoptotic body (fragmented nucleus). Scale bar represents 50 µm. (c) Exogenous albumin significantly increased OGD‐induced apoptosis (*n* = 3/group, *p* < 0.05). (d) *LRP2* siRNA decreased the expression of *LRP2* (*n* = 3/group, *p* < 0.05) DHK2 cells subjected to OGD in the presence of exogenous albumin demonstrated equivalent survival in control and megalin‐depleted conditions. (*n* = 4/group) Statistical analysis derived from one‐way ANOVA (a and b) or Student's *t*‐test (c and d)

To investigate the mechanism of albumin‐induced tubular injury in CRS‐1, we cultured HK2 cells, a human tubular epithelial cell line, with increasing albumin concentrations and subjected the cells to oxygen‐glucose deprivation (OGD). Under OGD conditions, HK2 cells demonstrated an albumin dose‐dependent reduction in survival (61.1 ± 1.44% in OGD with normal media control vs. 41.2 ± 5.54% in 25 mg/dl albumin vs. 24.0 ± 3.46% in 50 mg/dl albumin, *p* < 0.0001, *n* = 6/group, Figure 3a and b). An investigation of DNA aggregation in identically treated cells revealed that apoptosis accounted for the majority of epithelial cell death, suggesting that a cause of reduced HK2 cell survival was apoptosis (*p* = 0.001, *n* = 3/group, Figure 3c). These data indicate that albumin‐induced renal tubular apoptosis plays an important role of acute kidney injury in CRS‐1. On the other hand, the small interfering RNA‐induced inhibition of megalin, a well‐recognized endocytic receptor expressed in kidney epithelial cells, did not rescue HK2 cells from albumin toxicity, suggesting that exogenous albumin exacerbates acute cardiorenal syndrome by a megalin‐independent mechanism (Figure 3d and e). As cubilin may also mediate albumin uptake, we test whether cubilin knockdown altered albumin‐induced cell death in OGD; it did not (Figure S1).

## 4 DISCUSSION

The main finding of this study is that albumin plays a modulatory role in CRS‐1, augmenting tubular necrosis and loss of function. We found that CRS‐1 as modeled by CA/CPR induced severe albuminuria, which correlates with tubular epithelial cell death. Testing causality, we administered exogenous albumin in a clinically‐relevant fashion, which worsened AKI following CA/CPR. Mechanistic studies demonstrated that tubular epithelial cells take up excess albumin filtered in CRS‐1, and that albumin dose‐dependently induces tubular epithelial cell apoptosis. Together these findings support a deleterious role for albumin in CRS‐1. Our findings provide a mechanism for clinical observations, such as reported by Frenette et al, who retrospectively identified a dose‐dependent association between albumin administration and AKI in postoperative cardiac surgery patients (Frenette et al., 2014). Tantalizingly, the preoperative administration of albumin to hypoalbuminemic patients decreased the incidence of AKI in off‐pump cardiac surgery patients but not on‐pump cardiac surgery patients, suggesting that cardiac injury or excess exogenous albumin may be necessary for the development of albumin‐mediated AKI in CRS‐1 (Lee et al., 2016).

Supporting these findings, our *in vivo* microscopy studies demonstrated that CA/CPR induced a relative increase in glomerular filtration of albumin compared to that of very high molecular weight Dextran, and this leads to increased exposure and uptake of albumin in tubular epithelial cells. The mechanism by which this occurs is unclear. We previously reported that CA/CPR induces increased filtration of the charge‐neutral filtration marker Ficoll‐70, chosen because of its size similarity to albumin, suggesting that the size‐exclusion characteristics of the glomerular filtration barrier are altered by CA/CPR (Hutchens et al., 2012). Another group has reported that ischemia‐reperfusion altered filtration charge selectivity, leading to increased filtration of macromolecules, and it is possible that this mechanism explains our present observation (Rippe et al., 2006). We recently reported our observations made through the use of mass spectrometry of CRS‐1 failing to acutely alter the mass of endogenous albumin in glomerular filtrate, suggesting the current findings may stem from the increased mass of exogenous albumin, from decreased volume of urine with unchanged mass of albumin, or both (Tujjar et al., 2015). As use of albumin for resuscitation in cardiac surgery and critical care medicine is an active area of investigation, our results may inform ongoing and future studies, such as a proposed randomized double‐blinded clinical trial to determine the efficacy of cardiopulmonary bypass pump priming with albumin (Vlasov et al., 2020).

Lastly, our *in vitro* study demonstrated that cell death—primarily apoptosis—of tubular epithelial cells was increased and dose dependent on a high albumin concentration when incubated during the ischemia model. In this study, an increase of epithelial cell death was observed when incubated with 25 mg/ml albumin. We note that this concentration is higher than the expected concentration of albumin in the proximal tubule of normal rodents, valued from 0.02 to 1.0 mg/dl (Oken & Flamenbaum, 1971). However, the level of albuminuria observed after CA/CPR (Figure 1a), consistent with other such measurements in AKI (Hutchens et al., 2012), would lead to tubular concentrations greater than used in our cell culture experiments, and may therefore be potentially injurious *in vivo*. Similarly, the correlation observed between urine albumin concentration and kidney injury observed in our experiments may be seen as paralleling the dose‐dependency of albumin induced apoptosis *in vitro*; suggesting this correlation reflects causation rather than confounding. Overall, our data suggest that acute cardiorenal syndrome induces acute albumin exposure to tubular epithelial cells, which causes tubular epithelial cells apoptosis, and exogenous albumin loading can worsen this effect. The association between albuminuria and epithelial cell apoptosis is established in chronic kidney disease progression (Matsui et al., 2010), but to date, whether ischemia plays a role, and whether acute/transient albumin exposure is harmful has not been investigated. This novel finding may provide mechanism for the observed clinical and *in vivo* harm from administered albumin. As perioperative kidney injury is more common in patients with intraoperative hypotension (Walsh et al., 2013) and in those with CKD, these results may import the necessity of fluid resuscitation of patients with CKD.

Finally, we found that siRNA‐mediated megalin interference did not alter albumin's toxic effects on OGD‐exposed HK2 cells. Cubilin knock‐down also did not alter the effect of albumin. This suggests that a megalin‐ and/or cubilin‐ independent mechanism may be responsible for this effect. Other studies have characterized albumin transport (and that of other megalin ligands) by proximal tubular epithelium as being concentration‐dependent. Albumin transport is megalin dependent at low concentrations in diabetic nephropathy, but megalin‐independent at high concentration (Weyer et al., 2018). We speculate that high tubular concentrations of albumin during early acute cardiorenal syndrome may saturate the megalin‐ or cubilin‐ dependent albumin uptake mechanism, as suggested by our *in vivo* microscopy experiment, leaving a currently unidentified megalin‐independent uptake responsible for the observed cell death (Weyer et al., 2018). Mori et al previously reported increased albumin reabsorption in megalin knockout diabetic mice, which supports our megalin‐independent albumin reabsorption hypothesis (Mori et al., 2017).

Our study has some limitations. First, it should be noted that quantitative measurement of albumin filtration by *in* vivo 2‐photon microscopy has generated controversy. We therefore specifically evaluated albumin filtration relative to that of very high molecular weight Dextran, and we are confident in the finding of a relative increase due to CA/CPR. However, our study cannot be interpreted as a quantitative determination of change in albumin filtration. Second, experiments here are limited to *in vivo* and *in vitro* models; although our results are in accordance with clinical observations, humans may vary in their vulnerability to kidney injury from mice. Thirdly, we did not extensively evaluate the mechanism by which albumin induces apoptosis in renal tubular cells. Some candidate mechanisms have been reported by other groups. Activation of the CHOP pathway by BSA was reported as an inducer of tubular epithelial cell apoptosis (Ding et al., 2016; Wu et al., 2010). It is also reported that Brain abundant signal protein 1 (BASP1) mediates albumin‐induced apoptosis in tubular cells (Sanchez‐Niño et al., 2015). Additionally, mitochondria have been detected as a target of albumin‐induced proximal tubular apoptosis (Erkan et al., 2007). These might be involved in albumin mediated tubular apoptosis in CRS‐1. Lastly, we model CRS‐1 using CA/CPR, but CRS‐1 may be induced by several different kinds of cardiac insult, and results from myocardial infarction, acute heart failure, and cardiac surgery may vary as well.

In conclusion, administered albumin worsens AKI in CRS‐1, due to increased relative glomerular filtration and tubular apoptosis. Our findings provide mechanistic insight to recent clinical observations involving albumin resuscitation and may help guide interventions to improve patient outcomes.

## CONFLICT OF INTEREST

None.

## AUTHOR CONTRIBUTION

YF performed critical research, analyzed data, wrote and edited the manuscript. MI, RW, ZD designed and performed experiment. CS analyzed data. TG edited the manuscript. MH conceived, designed and performed experiments, analyzed data, wrote and edited the manuscript.

## Supporting information



Fig S1

Table S1
