# Supplementary figures and images for: Renal injury in cardiorenal syndrome type 1 is mediated by albumin
Source: Physiol Rep. 2022 Feb 12;10(3):e15173. doi: 10.14814/phy2.15173 (PMC8838648; doi:10.14814/phy2.15173)

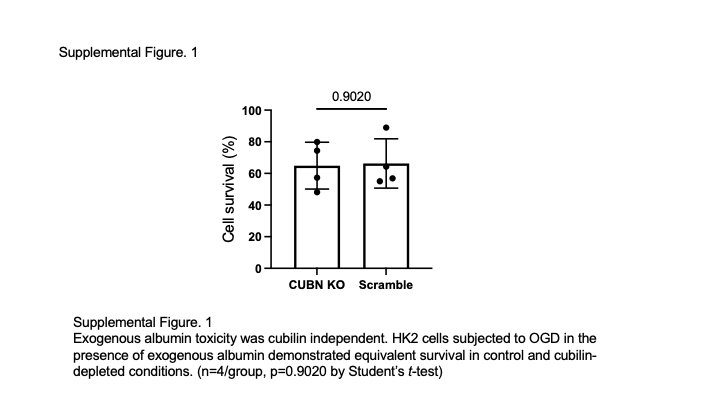

Supplement: Supplementary file 1 — Fig S1 [file PHY2-10-e15173-s002.tiff]
