# Supplementary material for: Renal injury in cardiorenal syndrome type 1 is mediated by albumin
Source: Physiol Rep. 2022 Feb 12;10(3):e15173. doi: 10.14814/phy2.15173 (PMC8838648; doi:10.14814/phy2.15173)
Supplement: Supplementary file 2 — Table S1 [file PHY2-10-e15173-s001.docx]

**Supplemental Table 1**

|  | **siRNA** | **Target gene** | **Locus** | **Sequence (5'-3')** | |  |
| --- | --- | --- | --- | --- | --- | --- |
|  | scramble siRNA |  |  |  | Not provided |  |
|  | LRP2 siRNA | *LRP2* | XM_011511184 | Sense | CAGGAGAACUUGCUCUGAATT |  |
|  |  |  |  | Antisense | UUCAGAGCAAGUUCUCCUGGT |  |
|  | CUBN siRNA | *CUBN* | NM_001081 | Sense | GGCAAUGGAUAUAUUUGCGTT |  |
|  |  |  |  | Antisense | CGCAAAUAUAUCCAUUGCCTT |  |

Supplemental table 1.

The sequences of siRNA used in the experiment is provided as above.
